# New method to measure interbreath intervals in infants for the assessment of apnoea and respiration

**DOI:** 10.1136/bmjresp-2021-001042

**Published:** 2021-12-10

**Authors:** Tricia Adjei, Ryan Purdy, João Jorge, Eleri Adams, Miranda Buckle, Ria Evans Fry, Gabrielle Green, Chetan Patel, Richard Rogers, Rebeccah Slater, Lionel Tarassenko, Mauricio Villarroel, Caroline Hartley

**Affiliations:** 1 Department of Paediatrics, University of Oxford, Oxford, UK; 2 Institute of Biomedical Engineering, Department of Engineering Science, University of Oxford, Oxford, UK; 3 Newborn Care Unit, Oxford University Hospitals NHS Foundation Trust, Oxford, UK; 4 Department of Ophthalmology, Oxford University Hospitals NHS Foundation Trust, Oxford, UK; 5 Department of Anaesthetics, Oxford University Hospitals NHS Foundation Trust, Oxford, UK

**Keywords:** respiratory measurement

## Abstract

**Background:**

Respiratory disorders, including apnoea, are common in preterm infants due to their immature respiratory control compared with term-born infants. However, our inability to accurately measure respiratory rate in hospitalised infants results in unreported episodes of apnoea and an incomplete picture of respiratory activity.

**Methods:**

We develop, validate and use a novel algorithm to identify interbreath intervals (IBIs) and apnoeas in preterm infants. In 42 preterm infants (1600 hours of recordings), we assess IBIs from the chest electrical impedance pneumograph using an adaptive amplitude threshold for the detection of breaths. The algorithm is refined by comparing its accuracy with clinically observed breaths and pauses in breathing. We develop an automated classifier to differentiate periods of true apnoea from artefactually low amplitude signal. We assess the performance of this algorithm in the detection of morphine-induced respiratory depression. Finally, we use the algorithm to investigate whether retinopathy of prematurity (ROP) screening alters the IBI distribution.

**Results:**

Individual breaths were detected with a false-positive rate of 13% and a false-negative rate of 12%. The classifier identified true apnoeas with an accuracy of 93%. As expected, morphine caused a significant shift in the IBI distribution towards longer IBIs. Following ROP screening, there was a significant increase in pauses in breathing that lasted more than 10 s (t-statistic=1.82, p=0.023). This was not reflected by changes in the monitor-derived respiratory rate and no episodes of apnoea were recorded in the medical records.

**Conclusions:**

We show that our algorithm offers an improved method for the identification of IBIs and apnoeas in preterm infants. Following ROP screening, increased respiratory instability can occur even in the absence of clinically significant apnoeas. Accurate assessment of infant respiratory activity is essential to inform clinical practice.

**Key messages**

- Can we improve the detection of apnoeas and respiratory activity in infants?
- We develop, validate and use a novel algorithm to identify interbreath intervals (IBIs) and apnoeas in infants, demonstrating improved sensitivity compared with the monitor-derived respiratory rate and clinically documented apnoeas.
- Respiratory disorders are common in preterm infants but without better measurement of respiratory activity, we will not fully understand the pathology and improve the treatment of these disorders.

## Introduction

Immature respiratory control in premature infants results in irregular patterns of breathing, with frequent pauses in breathing of variable duration.^1^ Apnoea (often defined as a pause in breathing lasting more than 20 s, or shorter if associated with a bradycardia or oxygen desaturation^2 3^) is a common pathology of prematurity, affecting more than 50% of preterm infants.^3^ These events can be life-threatening, result in reduced tissue oxygenation^4^ and may have long-term effects including reduced cognitive ability in childhood.^5 6^ Respiratory disorders are a common reason for admission to a neonatal unit.^7^ An infant’s respiratory activity may also be affected by pathologies including sepsis,^8^ pharmacological interventions including caffeine^9 10^ (administered as a treatment for apnoea of prematurity) and opioids^11^ (respiratory depressants) and painful clinically indicated procedures such as retinopathy of prematurity (ROP) screening.^12^ Despite the high prevalence of problems with respiratory control, clinical measurement of infant respiration is inadequate.^13 14^ While clinicians can rely on other physiological measurements to initiate the treatment of apnoeic episodes (eg, reductions in oxygen saturation and heart rate occur during prolonged pauses in breathing), self-resolving apnoeas may be missed^14^ and more subtle changes in respiratory activity will not be observed. Accurate assessment of respiration is essential to inform clinical practice and to understand respiratory development in health and disease.

Infants’ physiological data are continuously monitored in neonatal intensive care. Respiration is often computed by measuring changes in the electrical impedance of a patient’s thorax using the same electrodes that monitor the electrocardiograph (ECG). The use of impedance pneumography (IP) to assess respiratory function has known limitations, in particular susceptibility to noise, and was found by Lim *et al* to be less accurate than capsule pneumography;^15^ however, IP remains popular. Commercially available physiological monitors use built-in algorithms to process the IP signal and calculate the respiratory rate, often through the identification of peaks in the signal classified as breaths as a result of a specified amplitude threshold being exceeded.^16–18^ However, this approach is limited due to cardiac interference and artefacts caused by non-respiratory-related movements.^13 16 17^ Moreover, the manufacturers of many physiological monitors warn that their methods have yet to be validated for apnoea detection in infants.^16 17^ Research investigations have demonstrated the limitations of these monitors with high false-alarm rates and missed apnoeas.^13 14^ Lee and colleagues previously developed an algorithm to remove cardiac-frequency noise from the IP signal and demonstrated improved performance compared with built-in physiological monitor algorithms in the detection of neonatal apnoeas with a false positive rate of 5% and a false negative rate of 2.5%.^13^ However, they note that low amplitude signal related to factors such as poor electrode positioning or shallow breathing can be falsely identified as apnoeas; in a sample of 114 built-in apnoea monitor alarms, Lee reported that almost two-thirds were found to be false by clinicians (and similar rates have been found in other studies^19^). While their algorithm reduced this false alarm rate substantially to 37%,^13^ artefactually low amplitude signal remains a problem in apnoea detection. Additionally, accurate assessment of interbreath intervals (IBIs), and not just the identification of apnoeas as in the work of Lee *et al* is needed to gain a better understanding of the effects of pathology and interventions on respiration. For example, the assessment of more subtle changes in IBIs will improve classification of underlying pathology and may allow for the early detection and prediction of apnoeas.^20^

Here we develop a new method for identifying IBIs and apnoeas (defined here as pauses in breathing of at least 20 s) from an infant’s IP signal. We then use the algorithm to check its sensitivity to detect respiratory depression following morphine administration. Finally, we investigate changes in IBIs following ROP screening.

## Methods

### Study design

We designed, validated and tested our algorithm using three separate data sets. Data set 1 was used to determine the optimal threshold parameters for breath detection in the IP signals, by comparing the breaths identified with the algorithm to those manually annotated by clinical staff. Data set 2 was first used to verify that the parameters identified using data set 1 could detect pauses in respiration of at least 5 s. It was then used to develop and validate a classifier to detect true central apnoea as opposed to artefactually low amplitude signal. We then tested the algorithm, exploring its ability to identify morphine-induced respiratory depression, using a subset of data set 2. Finally, data sets 2 and 3 were used to evaluate changes in IBIs following ROP screening.

### Study participants

A total of 42 infants were included in this study. Data set 1 was collected as a subset of the MONITOR study.^21^ It comprises 181 sequences of approximately 40 breaths each (in total 7632 breaths), recorded from five preterm infants (postmenstrual age (PMA) at study range 30.6–34.3 weeks). Each breath was manually annotated by clinical staff in real time by visual observation of the infant. Data set 2 comprised physiological data collected during the Poppi trial, a single-centre, masked, randomised, placebo-controlled trial which investigated whether oral morphine was an effective and safe analgesic for procedural pain in premature-born infants.^11^ Physiological data were collected for 24 hours before and after the clinical procedure—a heel lance followed by ROP screening—in 30 infants (15 received morphine, 15 received placebo, PMA at study 34–39 weeks). Data set 3 is a previously unpublished data set of seven infants (PMA at study 30–37 weeks) whose physiological data were recorded before and after ROP screening. Further details for all studies are given in the online supplemental methods.

10.1136/bmjresp-2021-001042.supp1 Supplementary data

All data sets were collected at the Newborn Care Unit, John Radcliffe Hospital (Oxford University Hospitals NHS Foundation Trust, Oxford, UK). Written informed parental consent for all three data sets was gained. Approval was obtained from South Central Research Ethics Committee (REC) (13/SC/0597) for the MONITOR study, the Medicines and Healthcare products Regulatory Agency (MHRA) and Northampton REC (15/EM/0310) for the Poppi trial and from South Central REC (12/SC/0447) for data set 3. All studies conformed to the standards set by the Declaration of Helsinki.

### Physiological recordings

All infants were monitored using a Philips IntelliVue MX800 monitor, and physiological data were continuously downloaded from the monitor using ixTrend software (ixitos GmbH, Germany). Further details are given in the online supplemental methods.

### Breath detection from the IP signal

**Figure 1:**
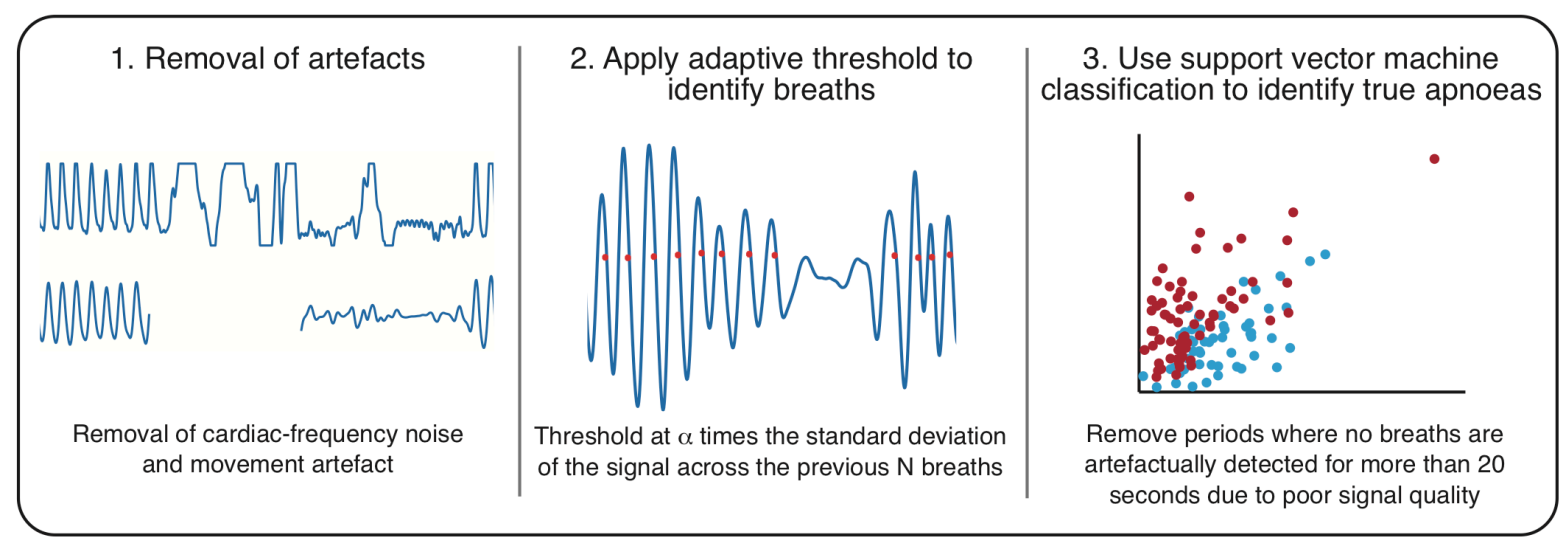
Schematic of the proposed algorithm for detection of interbreath intervals (IBIs) from the impedance pneumograph (IP) in infants.

The algorithm presented here to identify IBIs from the IP signal consists of three main steps (figure 1):

1. Removal of artefacts.
2. Application of an adaptive threshold to identify breaths.
3. Identification of true apnoeas using support vector machine (SVM) classification.

**Figure 2:**
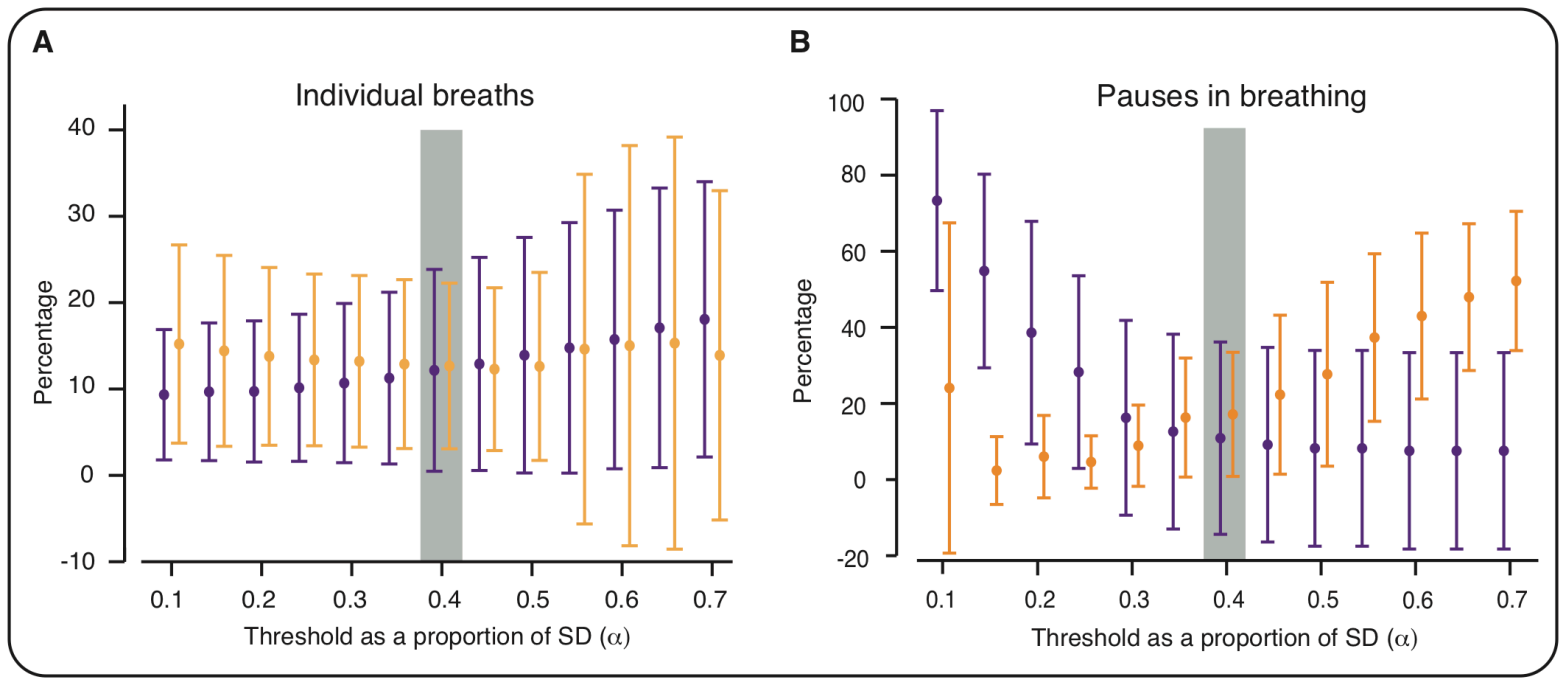
Optimising the threshold for breath detection. To optimise the threshold parameters, we investigated the performance of different threshold values (defined as a multiple (α) of the SD of the IP signal across the previous N breaths) to identify individual breaths and pauses in breathing. Figures show the percentage of false positives (orange) and false negatives (purple) for different values of α (with N=15). (A) Values calculated by comparing algorithm-identified breaths with breaths manually annotated at the time of the recording by visual observation (data set 1). (B) Values calculated by comparing algorithm-identified pauses in breathing with pauses (of at least 5 s) manually annotated by two investigators (first hour of recording in 15 infants from data set 2). Error bars indicate mean and SD (across the recordings). Values are jittered on the X-axis so that false positive and false negative bars do not overlap. Grey shading indicates selected threshold parameters; with these parameters (α=0.4, N=15), there was the optimal balance between the percentages of false positives and false negatives in the identification of individual breaths (A). These parameters also achieved a good balance between false positives and negatives in the identification of pauses in breathing (B).

The code for this algorithm is available from https://gitlab.com/paediatric_neuroimaging/identify_ibi_from_ip.git. For further details of all parts of the algorithm, see the online supplemental methods, figures 1,2 and tables 1,2. Briefly, first, the IP signals were filtered to remove artefacts not related to respiration, for example, large-amplitude changes caused by movements of the infant, and cardiac-frequency noise; the filtering process also zeroed the IP signals. Second, individual breaths were identified from the IP signal as the point at which an adaptive threshold is crossed (an adaptive threshold, ie, one that is updated across the recording,^22–24^ was used to account for changes in the amplitude of the signal for a variety of physiological and non-physiological reasons, such as shallow breathing and changes in the electrode and infant positioning). We identified the optimal threshold parameters for breath detection by comparing the breaths detected by the algorithm for different parameters with recordings where individual breaths were annotated in real time by a clinical member of staff visually observing the infant’s breathing (data set 1). The optimal parameters were chosen to be values which achieved the best compromise between the percentages of false positives and false negatives. We then verified that these parameters were also suitable for detection of pauses in breathing with a duration greater than 5 s by comparison of pauses in breathing detected by the algorithm with those that were retrospectively identified by two investigators (data set 2, first hour of recording, in 15 infants).

Finally, a linear SVM classifier was used to identify true central apnoeas (defined here as IBIs≥20 s) as opposed to artefactually low amplitude signal. The model input features are the magnitude (root-mean-square) of the filtered IP signal during the apnoea, in the 10 s prior to the apnoea, and in the 10 s after the end of apnoea, and the change in heart rate and oxygen saturation in the 60 s from the onset of the apnoea. The model was trained and tested using labels (true apnoea/false alarm) provided by two investigators for all potential apnoeas identified in data set 2 (training set, 15 infants who received morphine, test set, 15 infants who received placebo); 24% of potential apnoeas were classed differently by the two investigators and so were not included in the analysis.

### Performance of apnoea identification

To compare the accuracy of our approach with the accuracy of the current standard, all periods where the monitor-derived respiratory rate reached 0 were viewed by two investigators (see online supplemental material) and rated according to whether the investigator thought this period was a true central apnoea or a false alarm (90% inter-rater agreement occurred here). To compare with Lee *et al*, all episodes of apnoea accompanied by bradycardia (<100 bpm) and oxygen desaturation (<80%) detected by the algorithm were compared with investigator ratings to calculate the false-positive rate. To calculate the false-negative rate, all episodes of bradycardia (<100 bpm for at least 15 s) were identified in the recordings. Those that were not accompanied by a pause in breathing of at least 5 s (IBI >5) identified by the algorithm were rated according to whether the investigator thought a pause (true positive) in breathing occurred during this episode.

### Comparison with medical records

The time of apnoeas identified by our algorithm was compared with apnoeas documented in each infant’s medical records and nursing observation charts, specifically on the apnoea/bradycardia/desaturation chart (the term medical records is used to describe both in the rest of the paper). Apnoeas are documented if the clinical/nursing staff observe the infant having an episode of apnoea along with a description of how they were resolved, that is, self-resolving, requirement for increased oxygen, requirement for stimulation or requirement for resuscitation.

### Use of the algorithm to evaluate respiratory depression following morphine administration

We tested the algorithm by examining the changes in the IBI distribution following morphine administration in the 15 infants in data set 2 who received morphine. In the Poppi trial, we previously demonstrated a significant decrease in the respiratory rate (recorded on the monitor) in the morphine-treated infants compared with the placebo-treated infants, with a peak decrease approximately 2.5 hours following drug administration.^11^ We examined the IBI distribution in the 1 hour period prior to drug administration and the 1 hour period after the clinical procedure (from the end of the ROP screening, on average 1.3–2.3 hours after drug administration), and calculated the mean, median and SD of the IBI distributions, the proportion of IBIs longer than 5 s and the proportion of IBIs longer than 10 s (time periods commonly used to assess pauses in breathing^2^). We compared this with the mean monitor-derived respiratory rate calculated for the same periods.

### Use of the algorithm to evaluate changes in IBIs following ROP screening

We used the algorithm to investigate changes in the IBI distribution following ROP screening in a total of 22 infants—the 15 infants who received placebo in data set 2 and the seven infants in data set 3. In the placebo-treated infants, we compared the 1-hour period prior to placebo administration with the 1-hour period after the clinical procedure. In data set 3, we similarly compared the 1-hour after ROP screening with the 1-hour period 2.3–1.3 hours prior to ROP screening. We also compared the 12-hour period before and after ROP screening in the subset of 19 infants with at least 12 hours of recording before and after ROP screening.

### Statistical analysis

All data analysis was undertaken with MATLAB 2019b (MathWorks, USA). Model performance of the SVM classification was assessed with accuracy, false-positive rate, false-negative rate and Matthew’s correlation coefficient (MCC) using leave-one-subject-out cross-validation in the training set and independently in the test set using the model constructed from all infants in the training set. Differences in the IBI distribution and mean respiratory rate before and after morphine administration and ROP screening were compared using paired non-parametric t-tests with statistical significance assessed using permutation testing (10 000 random permutations) performed using FSLs PALM software.^25^ P values were adjusted for multiple comparisons using Hochberg’s method in R (The R Project for Statistical Computing).

### Patient and public involvement

A parent focus group, organised in collaboration with the charity SSNAP (Supporting the Sick Newborn and their Parents, a local charity based on the Newborn Care Unit at the John Radcliffe Hospital, Oxford), was held to discuss the Poppi Trial (data set 2) prior to the trial starting.

## Results

### Optimising the adaptive threshold

**Figure 3:**
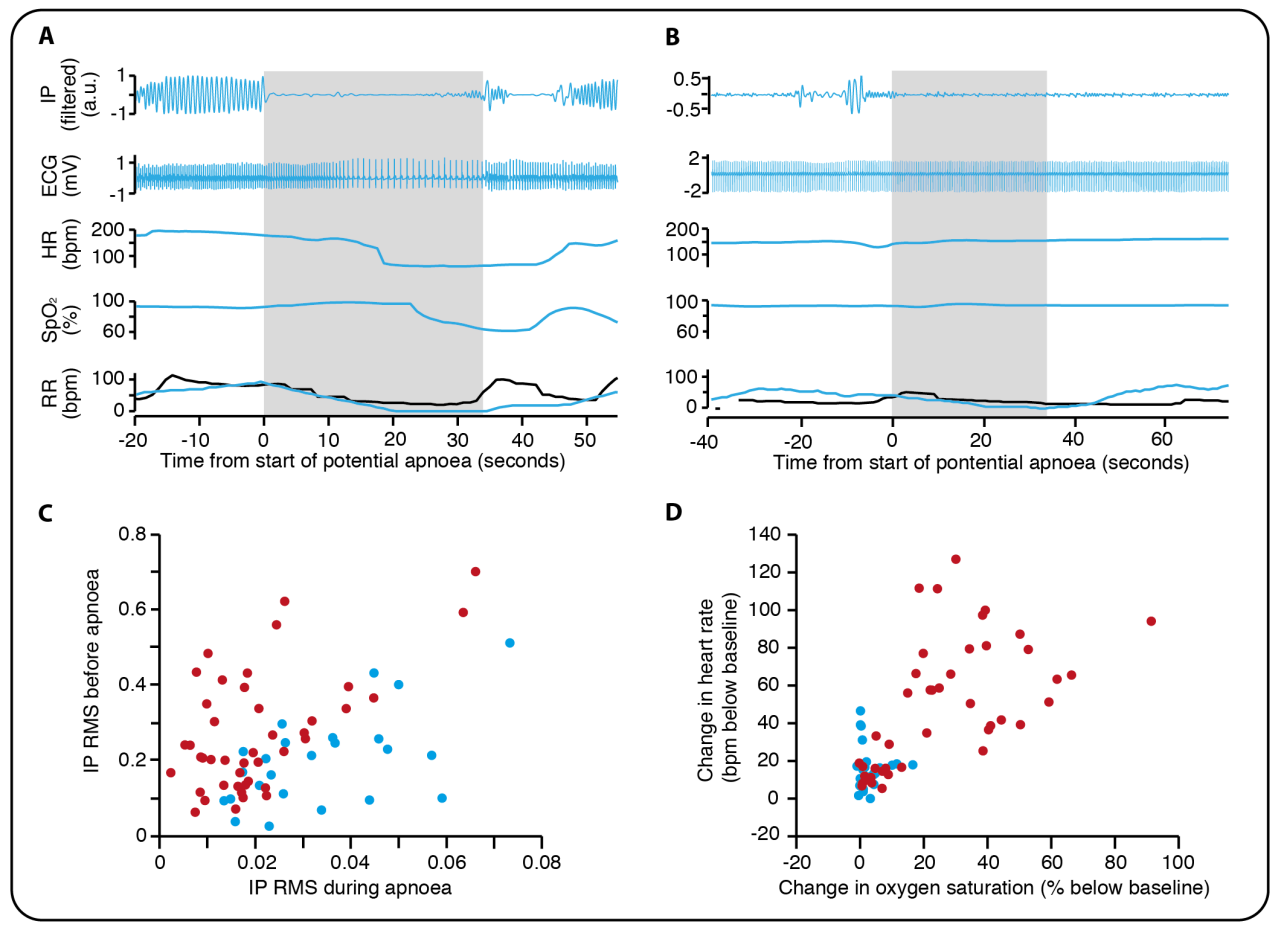
Using support vector machine classification to identify true apnoeas. (A) An example of a pause in breathing lasting longer than 20 s identified as a true apnoea. IP, the electrical impedance pneumograph after filtering to remove cardiac-frequency noise and movement artefact. HR, heart rate in beats per minute. SpO_2_, oxygen saturation. RR, respiratory rate in breaths per minute, recorded by the infant’s patient monitor (black) and calculated using our algorithm (blue). Note that the RR does not reach zero on the infant’s patient monitor and so this episode does not lead to a monitor apnoea alarm. Grey shading indicates the period during which no breaths were detected by our algorithm. (B) A potential apnoea initially detected by the algorithm but classified by investigators as a false alarm. (C) The root mean square (RMS) of the IP signal before and during the apnoea (see Methods for further details). Red circles indicate episodes classified by both investigators as true apnoeas, and blue circles are those episodes classified by both investigators as false alarms. (D) Change in oxygen saturation and heart rate for true apnoeas (red) compared with false alarms (blue).

A threshold of 0.4 times the SD of the filtered IP signal for the 15 previous breaths provided a good compromise between the false-positive and false-negative rates of breath detection (figure 2A, online supplemental results, figure 3). At this threshold, a mean (across all recordings in data set 1) of 12% of the manually annotated breaths were missed by the algorithm (false negatives), and 13% of breaths detected by the algorithm were false positives.

We examined whether these threshold parameters could also accurately identify pauses in breathing of at least 5 s. Using the same parameters, 13 pauses out of the 162 identified by both investigators were missed by our algorithm (false-negative rate: 8%) and 44 pauses out of the 229 identified by the algorithm were not identified by either investigator (false-positive rate: 19%). Varying the parameters confirmed that those selected achieved a good balance between false positives and false negatives (figure 2B).

### Optimising apnoea detection using machine learning

Applying the adaptive threshold to all recordings from data set 2 identified a total of 164 potential apnoeas. Of these episodes, 68 (41%) were classified by both investigators as true apnoeas and 57 (35%) were classified by both investigators as false alarms (no agreement for 39 (24%) episodes). This already represents a major improvement in detection rate from the monitor-derived respiratory rate—of the 71 occasions for which the monitor-derived respiratory rate reached a value of 0 breaths per minute, two episodes were classified by both investigators as true apnoeas (3%) and 62 (87%) were classified by both investigators as false alarms.

An SVM classifier was trained to distinguish between episodes detected by the adaptive threshold and classify them as either true apnoeas or false alarms (examples shown in figure 3A, B). In the training set (15 infants), using features derived from the IP signal alone (figure 3C, online supplemental methods), the classifier had an accuracy of 75% in the detection of true apnoeas (MCC=0.49, 62% of 69 episodes in the training set were true apnoeas). Including additional features related to the change in oxygen saturation and heart rate as inputs to the classifier and retraining it on the same training set increased the accuracy to 87% (MCC=0.74, false-positive rate=5%, false-negative rate=16%, figure 3D). Applying the best classification model to the test set gave an accuracy of 93% (MCC=0.87, false-positive rate=14%, false-negative rate=0%, 25 of 56 episodes in the test set were true apnoeas), validating the model in this independent data set.

For comparison with Lee *et al*,^13^ we performed three analyses. First, we assessed the performance of our algorithm for the detection of apnoeas with co-occurring bradycardia and oxygen desaturation. Of 26 such episodes (in both training and test set) detected by our algorithm, all were classified as true apnoeas by both investigators (0% false-positive rate). Second, we assessed the performance of our algorithm for the detection of pauses in breathing associated with bradycardias. A total of 109 episodes of bradycardia occurred in our data. Of the 13 episodes of bradycardia where a pause in breathing was not detected by our algorithm, only three were thought to be associated with pauses in breathing (3% false-negative rate) by the investigators. Finally, of the 62 false alarms where the monitor-derived respiratory rate reached a value of 0 breaths per minute, 3 (5%) were detected as apnoeas using the adaptive threshold alone. After applying the SVM classifier, none of these were detected as apnoeas by our algorithm (0% false alarm rate).

### Comparison with medical records

Of the 60 true apnoeas identified by our method, 88% were not recorded in the medical records. During the recording period, a total of 24 apnoeas were recorded in the medical records, the majority of which were associated with an IBI of at least 10 s; however, four events were not associated with a prolonged pause in breathing detected by the algorithm but instead with a prolonged loss of signal due to artefacts. We hypothesise that such artefacts were caused by clinical intervention in response to the apnoea.

### Use of the algorithm to evaluate respiratory depression following morphine administration

**Figure 4:**
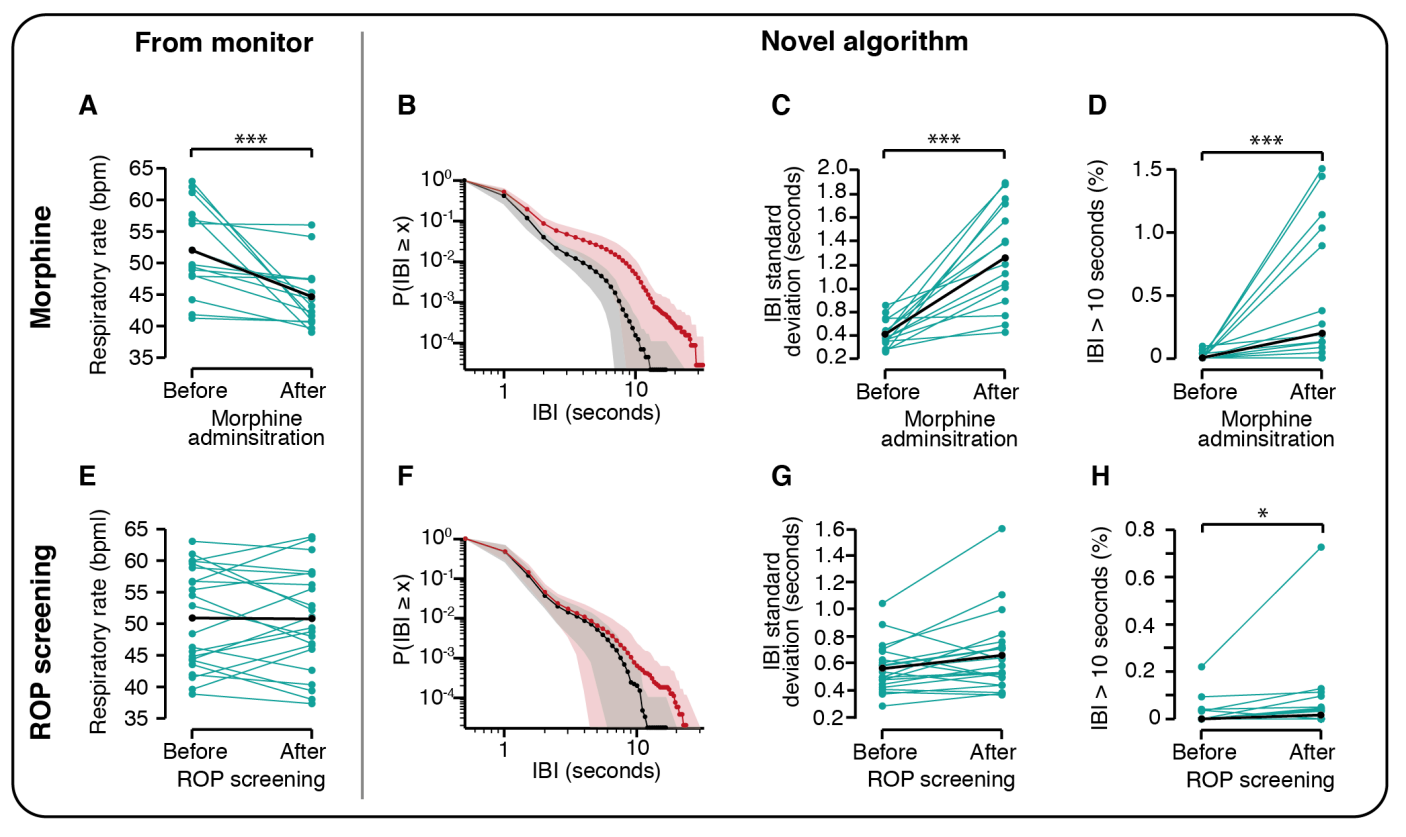
Interbreath intervals are altered by morphine administration and following ROP screening. (A–D) Respiratory rate and interbreath intervals (IBIs) in the 1-hour period prior to morphine administration compared with a 1-hour period after morphine administration (the 1-hour period immediately following ROP screening, approximately 1.3–2.3 hours after morphine administration) in the 15 infants who received morphine in the Poppi clinical trial. (E–H) Respiratory rate and IBIs 1 hour before and after ROP screening in 22 infants. (A, E) Mean respiratory rate from the infants’ patient monitor. (B–D, F–H) Metrics calculated using the novel algorithm proposed in this paper to identify the IBIs. Black lines and points indicate the group mean (A, C, E, F) or median (D, H). (B) IBI distribution in the 1-hour period prior to (black) compared with 1.3–2.3 hours after morphine administration (red). (F) IBI distribution in the 1-hour period before (black) and after (red) ROP screening. Y-axis indicates the probability of an IBI of duration greater than or equal to the X-axis value. Dotted line indicates the mean and shaded area the SD. (*p<0.05, **p<0.01, ***p<0.001, p-values corrected for multiple comparisons). ROP, retinopathy of prematurity.

As expected, there was a significant decrease in the monitor-derived respiratory rate following morphine administration (p=0.0004, non-parametric permutation t-test corrected for multiple comparisons, n=15, table 1, figure 4A, n=15). This was reflected in the IBI distribution, which showed a clear shift in the distribution towards longer IBIs following morphine administration (figure 4B), and significant differences in all IBI metrics assessed (figure 4C, D, table 1).

**Table 1:** Changes in interbreath intervals following morphine administration and ROP screening

	Mean before	Mean after	t-statistic	Uncorrected p value	Corrected p value
Morphine (n=15 infants)					
Mean respiratory rate (bpm)	52.01	44.66	−3.54	0.0001	0.0004***
Mean IBI (s)	1.07	1.34	5.18	0.0001	0.0004***
Median IBI (s)	0.93	1.03	3.96	0.0012	0.0012**
SD IBI (seconds)	0.61	1.26	5.86	0.0001	0.0004***
IBI >5 s (%)	0.56	2.54	4.17	0.0004	0.0008***
IBI >10 s (%)	0.02	0.49	3.39	0.0002	0.0006***
ROP screening (n=22 infants)					
Mean respiratory rate (bpm)	51.07	50.92	−0.14	0.89	0.89
Mean IBI (s)	1.09	1.12	1.32	0.20	0.81
Median IBI (s)	0.97	0.98	0.25	0.84	0.89
SD IBI (s)	0.56	0.66	2.45	0.021	0.10
IBI >5 s (%)	0.49	0.63	1.14	0.28	0.83
IBI >10 s (%)	0.02	0.06	1.82	0.0039	0.023*

Comparison of the respiratory rate (recorded by the patient monitor) and interbreath interval (IBI) distribution 1 hour before and after morphine administration and 1 hour before and after ROP screening. The table indicates the mean across all infants in each group, and the t-statistic and p-values for each comparison (permutation test). P-values were corrected for multiple comparisons using Hochberg’s method (*p<0.05, **p<0.01, ***p<0.001).

ROP, retinopathy of prematurity.

### Use of the algorithm to evaluate changes in IBIs following ROP screening

There was a shift in the IBI distribution in the 1 hour following ROP screening towards longer IBIs (figure 4F), with a significant increase in the proportion of IBIs longer than 10 s (p=0.023, figure 4H, table 1, n=22). This was not reflected by a change in the monitor-derived respiratory rate (p=0.89, figure 4E, table 1). Moreover, there was a significant increase in the proportion of IBIs longer than 10 s in the 12 hours after ROP screening compared with the 12 hours before (p=0.037, t-statistic=1.77, n=19). No apnoeas were recorded in the medical records in the 12 hours before or after ROP screening for any of these infants. Infant demographics are shown in online supplemental table 3.

## Discussion

We developed a new algorithm to detect IBIs from the IP signal in infants. Following the removal of cardiac artefact from the IP signals using a method introduced in Lee *et al*^13^ we used an adaptive amplitude threshold to identify individual breaths, validating the threshold by comparison with visually identified breaths and pauses in breathing. Previous studies have reported that signals with low amplitude due to poor electrode placement or shallow breathing can be erroneously detected as episodes of apnoea. To overcome this problem, we used machine learning to identify true apnoeas from periods of artifactually low amplitude. We tested our algorithm by investigating changes in IBIs following morphine administration, observing a clear shift in the IBI distribution consistent with the reduction in respiratory rate seen on the infants’ patient monitors. Finally, we used our algorithm to investigate changes in IBIs following ROP screening. We observed a significant shift in the IBI distribution following ROP screening which was not reflected by a change in the monitor-derived respiratory rate. This demonstrates the increased sensitivity of our method in detecting changes in respiratory activity, compared with the monitors and highlights the increase in physiological instability in infants following ROP screening.

Premature infants are born with immature cerebral and respiratory function compared with term-born infants, and consequently have a higher incidence of respiratory disorders. Current inadequacies in the measurement of respiration in infants leads to missed opportunities to better understand respiratory development and could potentially lead to suboptimal clinical treatment. For example, caffeine therapy, given for apnoea of prematurity, is stopped in infants between 33 and 35 weeks PMA if the infant appears clinically stable.^26^ However, in 10% of infants, caffeine treatment is restarted,^27^ which may suggest that caffeine was withdrawn too early, exposing the infants to the adverse consequences of lack of treatment. We found that 88% of apnoeas identified using our algorithm were not recorded in the medical records, consistent with previous results highlighting the inaccuracies in clinical documentation of apnoea.^14 28 29^ While there are multiple reasons why apnoeas may not be recorded accurately in clinical observations, including the under-recognition of apnoeas that come within periods of periodic breathing, this substantial increase in the number of apnoeas identified using our algorithm demonstrates the potential for improving apnoea detection. Improved measurement of respiration is essential to optimise clinical treatment of apnoea and could enhance treatment for other clinical conditions or procedures which alter respiration.

Many drugs will alter infants’ physiology. Our results confirm the applicability of the algorithm to analyse morphine-related respiration depression. Using this approach to investigate respiratory changes in relation to other drugs commonly prescribed in neonatal care may enhance our understanding of pharmacodynamics. Additionally, analysis of physiological recordings may be useful to develop predictive models to tailor individualised care.^30 31^ We recently showed in a post-hoc analysis of the morphine-treated infants in the Poppi trial that we could predict the risk of adverse cardiorespiratory effects in individual infants from their baseline physiological stability.^32^ To date, measures of respiration are often not included in the development of predictive tools, which is likely due to the relatively poor quality of the currently available measurement tools.^31^ Here we provide a more accurate measure of IBIs, which will allow for more complex metrics, such as respiratory rate variability, to be computed.

ROP screening, an eye examination that is thought to be painful and distressing for infants,^33^ has previously been shown to increase the rate of apnoea in the 24–48 hours following the screen from clinical chart review.^12^ In an exploratory analysis, we demonstrated a significant increase in the proportion of IBIs longer than 10 s in the 1-hour and 12-hour periods after ROP screening, which was not reflected by a change in the monitor-derived respiratory rate. This demonstrates the improved sensitivity of our method for identifying changes in respiratory activity and suggests that even those infants without clinically significant apnoeas may still experience changes in respiratory activity with a shift towards longer IBIs. Further research in a larger cohort across a wider age range is needed to explore the relationship between an infant’s respiratory activity following ROP screening and changes with age. Identifying older infants that are at risk of physiological instability after ROP screening would be particularly important for those ex-premature infants who have ROP screening in outpatient clinics and may benefit from observation before leaving hospital.^34^

To remove cardiac-frequency noise from the IP signals, we used the approach of Lee and colleagues,^13^ which we modified (online supplemental table S2) predominantly due to the poor performance of the ECG R peak detection used by Lee *et al* in our data. Our algorithm had similar rates of false positives and negatives in the identification of apnoeas to those reported by Lee. Importantly, we also trained a classifier to identify true apnoeas compared with artifactually low amplitude signal; the classifier reduced the false alarm rate compared with using the adaptive threshold alone and to that reported by Lee and colleagues. Additionally, our algorithm used an adaptive threshold to identify individual breaths (with thresholds optimised with and without the prior removal of cardiac-frequency noise). Thus, unlike the algorithm of Lee, our algorithm can be used both in the identification of apnoea and also to examine changes in the pattern of IBIs of an infant.

By using an adaptive threshold which we validated for infants, our algorithm performed substantially better than the monitor derived respiratory rate. However, limitations of this study are the relatively small sample size and narrow age range of the infants included (from 30 to 39 weeks PMA). Further validation should be carried out in younger infants. Moreover, this method identifies central apnoea; it cannot detect obstructive apnoea—alternative measures, such as nasal air flow, are needed to detect these events. Additionally, apnoea that necessitates intervention by clinical staff may not be detected or the reported duration may be shorter than the true duration of the episode as interventions are likely to lead to large artefacts in the IP signal. While this is not a problem for clinical management, as the infant is receiving the appropriate clinical intervention to support their breathing, this should be taken into account in research studies so that apnoeas are not missed in the analysis.

In summary, despite the common occurrence of respiratory pathology in preterm infants, current methods used to measure respiration are inadequate. We developed a new method to measure respiration in infants, demonstrating the improved sensitivity of the method compared with current standards; the increased sensitivity provided by our algorithm could aid clinical teams in the care of infants. Furthermore, we identified a significant increase in respiratory instability in infants following ROP screening. A better understanding of respiratory activity in infants is critical to improve neonatal care.

## Data Availability

Data are available upon reasonable request. The code for the algorithm developed in this paper is available from https://gitlab.com/paediatric_neuroimaging/identify_ibi_from_ip.git.
